# Organic Electrochemical Transistors as Versatile Analytical Potentiometric Sensors

**DOI:** 10.3389/fbioe.2019.00354

**Published:** 2019-11-22

**Authors:** Isacco Gualandi, Marta Tessarolo, Federica Mariani, Domenica Tonelli, Beatrice Fraboni, Erika Scavetta

**Affiliations:** ^1^Dipartimento di Chimica Industriale ‘Toso Montanari’, Università di Bologna, Bologna, Italy; ^2^Dipartimento di Fisica e Astronomia, Università di Bologna, Bologna, Italy

**Keywords:** OECT, potentiometric sensors, chloride, chemical sensors, PEDOT:PSS

## Abstract

Potentiometric transduction is an important tool of analytical chemistry to record chemical signals, but some constraints in the miniaturization and low-cost fabrication of the reference electrode are a bottleneck in the realization of more-advanced devices such as wearable and lab-on-a-chip sensors. Here, an organic electrochemical transistor (OECT) has been designed with an alternative architecture that allows to record the potentiometric signals of gate electrodes, which have been chemically modified to obtain Ag/Ag_n_X interfaces (X = Cl^−^, Br^−^, I^−^, and S^2−^), without the use of a reference electrode. When the OECT is immersed in a sample solution, it reaches an equilibrium state, because PEDOT:PSS exchanges charges with the electrolyte until its Fermi level is aligned to the one of Ag/Ag_n_X. The latter is controlled by X^n−^ concentration in the solution. As a consequence, in this spontaneous process, the conductivity of PEDOT:PSS changes with the electrochemical potential of the modified gate electrode without any external bias. The sensor works by applying only a fixed drain current or drain voltage and thus the OECT sensor operates with just two terminals. It is also demonstrated that, in this configuration, gate potential values extracted from the drain current are in good agreement with the ones measured with respect to a reference electrode being perfectly correlated (linear slope equal to 1.00 ± 0.03). In the case of the sulfide anion, the OECT performance overcomes the limit represented by the Nernst equation, with a sensitivity of 0.52 V decade^−1^. The presented results suggest that OECTs could be a viable option to fabricate advanced sensors based on potentiometric transduction.

## Introduction

**Figure 1:**
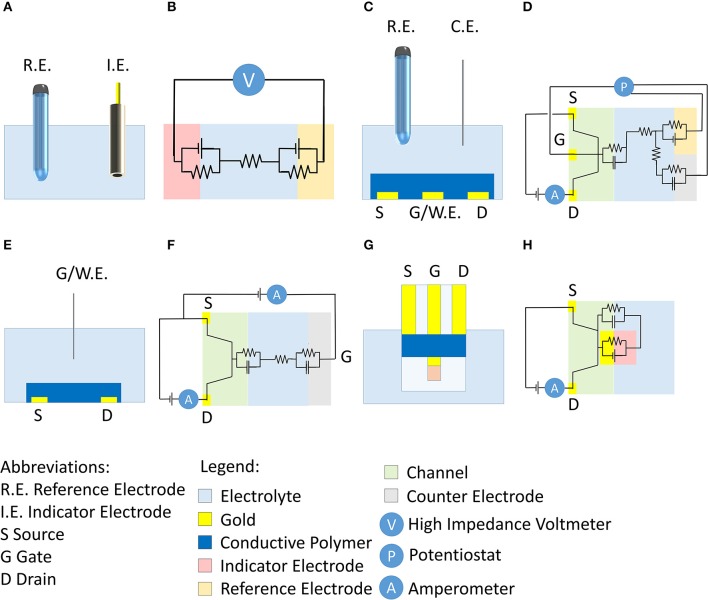
**(A)** Conventional setup of a potentiometric measurement and corresponding electrical circuit **(B)**. **(C)** Scheme of the OECT setup proposed by Wrighton's group (Kittlesen et al., 1984; Paul et al., 1985) and corresponding electrical circuit **(D)**. **(E)** Scheme of OECTs that are commonly used in literature and corresponding electrical scheme **(F)**. **(G)** Scheme of here-proposed OECT for potentiometric measurements and corresponding electrical scheme **(H)**.

Potentiometric sensors are important tools in analytical chemistry to quantify the concentration of chemical species in solution, however, some constraints hinder their low cost-fabrication, miniaturization, and thus their reliable use in some emerging fields such as wearable and lab-on-chip technologies (Sophocleous and Atkinson, 2017; Parrilla et al., 2019). A potentiometric measurement is performed with a high impedance voltmeter that measures a voltage difference in absence of a current flow between an indicator and a reference electrode, both dipped in the same solution (Skoog et al., 1992; Figure 1A). The circuit is very simple and does not require other elements (Figure 1B). The response is controlled by the activity of the species that is quantified during the analysis, as ruled by Nernst equation. Among these sensors, the glass electrode for pH measurement has remained the gold standard for more than a century (Haber and Klemensiewicz, 1909; Skoog et al., 1992; Harris, 2016). Moreover, other potentiometric sensors have been developed for the detection of various chemical species such as fluoride and calcium, and these devices are now available on the market (Light and Cappuccino, 2009). Potentiometric measurements can be applied to very diverse fields in analytical chemistry due to their simplicity and fast response time, even if their use is limited by the difficulty in the fabrication of membranes able to selectively detect the target analyte.

The convergence of new technological progresses, mainly based on informatics and telecommunications, is dramatically modifying the production and control systems, while the novel concepts of Industry 4.0 and internet of things are becoming popular. There is an outstanding demand for fast data collection and elaboration that strongly renews the interest for chemical sensing (Potyrailo, 2016). Therefore, the research efforts are now focusing on the development of analytical tools that can be used in real time, without the need of laboratory facilities and expert users. These devices should be portable (Cuartero and Crespo, 2018) or even wearable (Tessarolo et al., 2018; Parrilla et al., 2019) in order to acquire information *in situ* that can range from the home-bed of a patient to a polluted site. Consequently, the miniaturization and integration of chemical sensors in every-day-objects play a key role to allow a non-invasive monitoring or to produce electronics boards, which act as a full laboratory on a chip. Potentiometric sensors take advantage of a direct transduction of the chemical signal into an electrical one with a very low energy consumption associated to the read-out of the signal. In spite of that, the miniaturization and low-cost fabrication of reference electrodes are the actual bottleneck in the development of reliable potentiometric sensors for wearable, portable and lab-on-a-chip technologies (Sophocleous and Atkinson, 2017). A possible solution is the use of a screen-printed reference electrode, but the literature (Sophocleous and Atkinson, 2017) reports that reliable Ag/AgCl screen-printed reference electrodes are still only at a primal level. Furthermore, the potential of such electrodes is not stable enough to allow their use in potentiometry, because the KCl out-diffusion and in-diffusion of ions from the electrolyte solution limit their stable lifetime (Bergveld, 2003; Sophocleous and Atkinson, 2017). Transistors have been exploited to measure potentiometric signals. In fact, ion selective field-effect transistor (ISFET) are usually classified as potentiometric sensors, as they measure an interface potential. ISFETs also require a reference electrode to work and, thus, they suffer the limitations above pointed out as to device miniaturization and fabrication (Bergveld, 2003).

In this scenario, Organic ElectroChemical Transistors (OECTs) show favorable features to address these limitations (Lin and Yan, 2012; Pappa et al., 2018; Rivnay et al., 2018). Typically, they are three terminal devices (gate, drain, and source) where the source and drain electrodes are electrically connected by a channel made of a thin film of a conducting polymer [typically poly(3,4-ethylenedioxythiophene) doped with polystyrene sulfonate, PEDOT:PSS]. Differently from field effect transistors (FET) where only a thin layer is involved in the change of conductivity upon gate action, in an OECT the semiconducting material as a whole changes its Fermi level and the charge carriers concentration. Consequently, OECTs exhibit high transconductance values that usually overrun the ones of other transistors, both based on organic and inorganic semiconductors (Rivnay et al., 2018). During transistor operation, a voltage is applied to the drain (V_d_) and the generated current (I_d_) that flows between drain and source (grounded) is measured. I_d_ can be modulated through the application of a voltage to the gate terminal (V_g_) that stimulates the occurrence of electrochemical reactions leading to a variation of the charge carriers concentration in the polymer. The gate electrode can be placed in different positions with respect to the channel. In the first articles describing OECTs, Wrighton's group (Kittlesen et al., 1984; Paul et al., 1985) proposed a channel that was in direct electrical contact with the gate biased with a potentiostat (Figures 1C,D). The potentiostat kept the desired potential difference between the gate and reference electrodes, while the current flew between the gate and counter electrodes through the electrolyte. The electrical circuit is shown in Figure 1D. Since the gate potential is ideally applied with respect to a reference electrode in such a case we use the symbol E_g_. The channel and gate, being in direct contact in this architecture, are at the same potential E_g_, and thus it is possible to directly control the charge carrier concentration in the conductive polymer. However, the configuration did not allow the chemical modification of the gate electrode that is usually exploited as transducer. For this reason, recent literature mostly proposes OECTs wherein the gate electrode is separated from the channel (Figure 1E). The OECT works without a reference electrode and, thus, the potentiostat is replaced by a source meter to polarize the gate electrode with respect to the source (grounded) (Figure 1F). The positive/negative V_g_ application repels/attracts cations that are pushed into/pulled off the conductive polymer in the channel. These processes induce the extraction/injection of charge carriers in the channel with a consequent oxidation/reduction of the semiconducting polymer. Since the solid-liquid interfaces at the channel and gate electrode do not exhibit a known electrochemical potential and no reference electrode is used in the OECT configuration, any information/control on the electrochemical potential is lost.

Since sensors based on OECT are usually fabricated without a reference electrode (Lin and Yan, 2012; Pappa et al., 2018; Rivnay et al., 2018), they can be produced by means of soft fabrication techniques, such as spin coating (Gualandi et al., 2015), roll-to-roll processing (Berggren et al., 2007) and ink-jet printing (Demelas et al., 2013; Wustoni et al., 2019), and thus they are easily integrated in real-life objects such as fabrics (Gualandi et al., 2016a), paper (Bihar et al., 2016), and plastic (Mariani et al., 2018). In addition, OECTs exhibit a high biocompatibility because they can be made of non-toxic materials which are also soft and lightweight and thus they can adapt to the mechanical features of biological tissues. In fact, flexible OECTs have been proposed for non-invasive monitoring of electrical signals inside the brain (Khodagholy et al., 2013). Moreover, the transistor configuration offers an intrinsic signal amplification (Gualandi et al., 2018a) that allows achieving a high sensitivity and/or the use of simpler read-out electronics. In recent published contribution (Gualandi et al., 2018a), it is showed that in the presence of a redox-active molecule, such as ascorbic acid, the Faradaic current that is at the basis of electrochemical signal transduction is generated by ascorbic acid oxidation and measured at the gate electrode. This process leads to a drain current variation that is higher than that recorded at the gate, thus demonstrating an enhanced response. When OECTs are used as chemical sensors, the transduction and selectivity features are usually obtained with a gate electrode that can interact with the analyte. Usually, the same chemically-modified electrodes exploited in amperometric and potentiometric sensors are used as gate electrodes of OECTs. OECTs endowed with a PEDOT:PSS gate electrode can be used to quantify ascorbic acid, dopamine, adrenaline and uric acid (Gualandi et al., 2015, 2016b) thanks to the same electrocatalytic processes reported for the amperometric sensors. When an OECT is endowed with a gate electrode modified with an appropriate enzyme, it can detect metabolites such as glucose (Bernards et al., 2008; Diacci et al., 2019; Wustoni et al., 2019) and ethanol (Bihar et al., 2017). Although the literature reports a lot of examples of OECTs where an amperometric transduction can be invoked to explain the operating principle, there are only a few examples of OECTs equipped with gate electrodes able to perform a potentiometric measurement. For example, chloride ions (Tarabella et al., 2010) and pH (Scheiblin et al., 2017) can be detected by OECTs wherein the gate electrodes are silver and platinum/iridium oxide, respectively, that are the same responsive materials as present in potentiometric sensors (Parrilla et al., 2019). Moreover, an ultra-sensitive protein detection can be accomplished by the measurement of the threshold potential of an OECT endowed with a gate electrode modified with a bio-receptor (Macchia et al., 2018). Bernards et al. (2008) issued the main model that explains OECTs' operation as sensors. It is based on a variation of the effective gate potential that is generated by a change of the solution electrochemical potential, which is in turn controlled by the analyte concentration. To the best of our knowledge, no attempt has been carried out until today to exploit an OECT for the measurement of an electrochemical potential.

This work proposes a novel strategy to record a potentiometric signal by means of an OECT, and consequently, without the use of a reference electrode. An alternative OECT architecture (Figure 1G) has been designed in order to have a gate electrode that: (i) is in direct contact with the channel in order to enable the measurement/control of the electrochemical potential of the conductive polymer. Thanks to this contact between the two elements, the two electrochemical interfaces have the same potential; (ii) can be chemically modified in order to perform the potentiometric transduction. The gate has been modified with an Ag/Ag_n_X (X = Cl^−^, Br^−^, I^−^, and S^2−^) layer to produce a second kind electrode, whose potential is controlled by the X^n−^ activity in solution. It is well-known that a second kind electrode is composed by a metal, in contact with one of its insoluble salt and a solution of the relative anion. The equilibrium that is established among different phases enables the electrode to have a fixed potential from a thermodynamic point of view, i.e., measured with respect to a reference electrode. Since the Ag/Ag_n_X electrode spontaneously reacts with the anions present in the solution, the gate potential is modulated without the use of an external potential source. Consequently, there is no need of a dedicated electrical connection for the gate bias, and an OECT architecture with only two electrical terminals can be proposed (Figure 1H), realizing a simpler set up compared to other transistor configurations. When Ag/AgCl is used, the proposed sensor can determine the Cl^−^ concentration that is relevant in sweat analysis, for instance in the diagnosis and monitoring of cystic fibrosis (Savant and McColley, 2019) and for the non-invasive evaluation of the body hydration level (Gao et al., 2016).

## Materials and Methods

### Chemicals

CLEVIOS™ PH 1000 suspension (PEDOT:PSS) was bought from Heraeus (3-glycidyloxypropyl)trimethoxysilane, dodecylbenzenesulfonic acid, potassium bromide, potassium iodide, potassium nitrate and potassium hydroxide were purchased by Sigma Aldrich. Potassium chloride was bought from Fluka. Ethylene glycol was obtained from Carlo Erba. Sodium hydroxide was purchased by Empura. All chemicals were of reagent grade or higher. The glass slides were obtained from Menzel-Gleaser. Polydimethylsiloxane (PDMS) was prepared using Sylgard 184 kit that was bought from Dow Corning.

### Apparatus

The electrochemical potentials were ideally applied with respect to a saturated calomel electrode (SCE) in a single compartment, three-electrode cell via a potentiostat (CH Instrument 660C). The gate electrode was connected to the working electrode output and a Pt wire was used as the counter electrode. It is worthy to note the difference between electrochemical potential and other applied voltage/potential. The electrochemical potential is measured or ideally applied with respect to a reference electrode, and as already stated, we used the symbol E to indicate it in this paper. This potential is strictly related to the thermodynamics of the different processes that can take place at the electrode surface. When the electrochemical potential is applied or measured at the gate electrode, the E_g_ symbol is used. Conversely, we use the symbol V to express a voltage that is applied/measured with respect to the ground or the source terminal, but it is not referred to an electrochemical interface with a reproducible and well-defined potential (measured with respect to a reference electrode).

During the transistor characterization a Keysight B2902 A source-meter unit applied the drain voltage, while the gate potential was applied by a potentiostat. The output and the transfer curves were recorded in 0.1 M KNO_3_ solution.

The sensing experiments were performed while the Keysight B2902 A equipment applied the V_d_ (−0.01 V) and measured the I_d_, while the gate and the channel were dipped in 0.1 M KNO_3_ and different amounts of the analytes were added to the electrolyte solution. In order to measure the gate electrochemical potential and compare it with the value estimated by the drain current, a reference electrode has been used.

### OECTs Fabrication

Gate, drain, and source contacts made of Cr/Au (50 nm) were deposited via thermal evaporation. The thickness of the Cr layer is 10 nm, while the Au one is 40 nm. PEDOT:PSS solution was prepared by adding 5% v/v ethylene glycol, 0.25% v/v dodecylbenzenesulfonic acid, and 1% v/v 3-glycidyloxypropyltrimethoxysilane to the CLEVIOS PH 1000 suspension. The mixture was filtered through a 1.2 μm cellulose acetate filter and spin coated between gold source and drain electrodes at 500 rpm for 3 s, and then annealed at 140°C for 1 h. The final thickness of the PEDOT:PSS channel was 800 nm. Afterward, the gate electrode of the transistor was modified with Ag/Ag_n_X (X = Cl^−^, Br^−^, I^−^, and S^2−^). To this aim, the bottom part of the gate electrode was carefully dipped in the electrodeposition solutions in order to avoid the contact between the electrolyte and the channel. The gate electrode was connected to the working electrode terminal of the potentiostat. Firstly, silver was deposited on the electrode by applying −0.2 V using 0.1 M AgNO_3_ as the electrodeposition solution. After washing with distilled water, the gate modification was completed by applying +0.6 V when the electrode was dipped in a 0.1 M solution of X^n−^ anion (X^n−^ is the analyte to be detected by the sensor). If the sensor was not used just after the preparation, it got dried. Before the use, the OECT was hydrated by soaking it in the electrolyte solution.

### Calculation of E_g_ From I_d_

The E_g_ values were calculated from the I_d_ values recorded during an experiment for Cl^−^ detection by exploiting the transfer curve as calibration plot of the transistor. In detail, the transfer curve was recorded before the chemical modification of the gate electrode with Ag/AgCl because the application of an E_g_ would cause a current flow that changes the gate chemical composition. After that, the gate electrode was modified with the procedure described in the paragraph 2.3 and the obtained OECT was used for the Cl^−^ detection. The sensing experiment was performed with the procedure described in the paragraph 2.2, and E_g_ was measured with respect to a reference electrode. The I_d_ values were extracted for every Cl^−^ addition. The transfer curve was approximated to a line in the range of the observed I_d_ values, where I_d_ was the y-axis and E_g_ the x-axis. The E_g_ values were calculated from I_d_ values by means of this line and were compared with the ones measured with respect to the reference electrode.

## Results

### OECT Characterization

**Figure 2:**
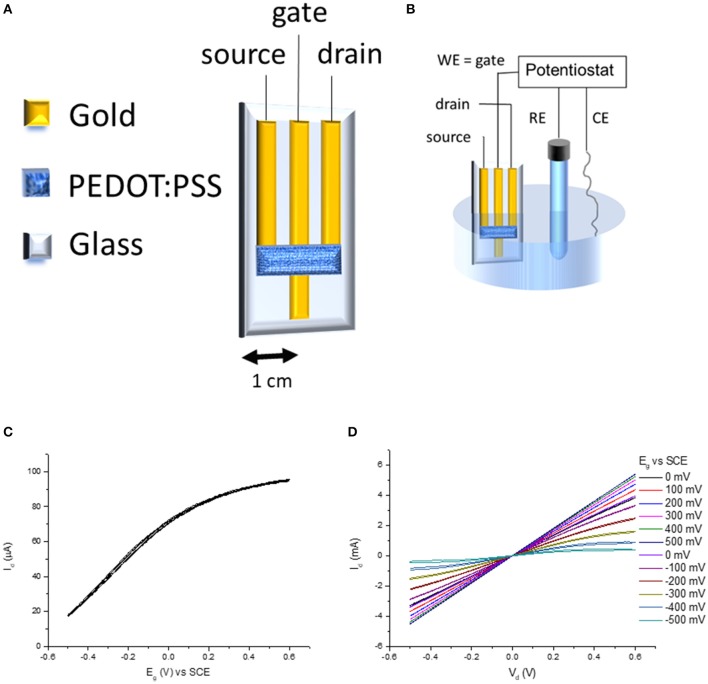
**(A)** OECT architecture, **(B)** experimental setup for acquiring transfer curve **(C)**, and output curves **(D)**.

The transistors were fabricated with a geometry that is ideally similar to the one proposed by Wrighton's group (Kittlesen et al., 1984; Paul et al., 1985) in the first articles describing OECTs, because the gate electrode is in electrical contact with the conductive polymer present in the channel (Figure 2A). The source and drain terminals are the outer gold tracks, while the gate terminal is the inner gold track. The gold track of the gate is longer than source and drain ones in order to allow for the electrodeposition of the sensing material without immersing the conductive polymer. The channel is the PEDOT:PSS film connecting the drain and the source terminal. Consequently, the gate terminal can control the electrochemical potential of the conductive polymer when the device is dipped in a solution and connected to a potentiostat (Figure 2B). In order to complete the transistor architecture, a source-meter unit applies a voltage between the drain and source terminals and records the generated current (I_d_). **Figure 2C** shows the transfer curve of the transistor. As above described, the gate electrode is in direct electrical contact with the channel. Consequently, the two elements reach an equilibrium state, and thus they have the same potential. When a positive voltage is applied to the gate, also PEDOT:PSS acquires a positive voltage and the cations are pushed out causing holes injection. From an electrochemical point of view, PEDOT:PSS is converted in its oxidized state according to the reaction (1):

$$\begin{array}{l} \text{Reaction}1:\text{PEDOT}+\text{Na}:\text{PSS}\to \text{PEDOT}:\text{PSS}+\text{N}{\text{a}}^{+}+{\text{e}}^{-} \end{array}$$

Since charge carriers are the oxidized centers of the polymer, the electrical conductivity of the polymer increases. On the other hand, if a cathodic/negative potential is applied to the gate, the reduction of the polarons and bipolarons takes place with a decrease of the charge carriers concentration, and thus of the source-drain current. The transistor behavior is in agreement with the observation of Wrighton's group (Kittlesen et al., 1984) which studied a transistor with a polypyrrole channel in direct contact with the gate electrode. The OECT output curves (Figure 2D) show the ability of the gate voltage to control the channel conductivity, demonstrating that the device works as a transistor.

Since E_g_ is applied with respect to a reference electrode, it is directly related to the Gibbs energy (ΔG) and thus to the thermodynamics of the system through the equation ΔG = −n F ΔE, where n is the number of electron involved in the redox reaction, F is the Faraday constant and ΔE is the potential measured between the electrodes (Bard and Faulkner, 2000). Since ΔG is connected to the reaction quotient Q_c_ by the relationship ΔG = −R T ln Q_c_ (where R is the ideal gas constant and T is the absolute temperature), it is possible to control the concentration of the oxidized and reduced forms at the electrochemical interfaces by the application of a potential.

In a simple molecular entity, the formal potentials of the first occurring oxidation and reduction processes are roughly correlated (less than the electrostatic interaction) to the energy levels of the HOMO and LUMO (Méndez-Hernández et al., 2013). Passing from simple molecules to conductive polymers, the E value controls the concentration of polarons and bipolarons (oxidized forms of the polymer), which are the charge carriers, and thus the doping degree. The electrochemical doping/de-doping processes can be investigated by cyclic voltammetry and it can be exploited to study the electronic band structure of the conductive polymer. In fact, the E values can be converted to the energy of vacuum state by simply summing a constant (Zoski, 2007). Therefore, the gate electrode directly controls the electrochemical potential E of the channel, and consequently the doping state of the polymer. Since in the configuration of the here-proposed transistor the geometry ideally does not affect the concentration of charge carriers, a beneficial effect on the robustness of the device can be observed when the OECT is used as a chemical sensor.

The equation that rules the operation of the transistor is the following (Bernards and Malliaras, 2007; Rivnay et al., 2015):

(1) $$\begin{array}{l} I_{d}\left(x\right)=\;I_{d}=W\;d\;e\;u\;\rho \left(x\right)\;\frac{dV\left(x\right)}{dx} \end{array}$$

Where W, d, μ, and e are the width, thickness of the channel, the mobility and the charge of the hole respectively and ρ(x) is the distribution of the charge carrier concentration in the channel. The potential V(x) is the voltage in function of position, and measured with respect to the ground.

However:

(2) $$\begin{array}{l} \frac{dV\left(x\right)}{dx}=\;\frac{dE\left(x\right)}{dx} \end{array}$$

because E and V differ by a constant.

Since PEDOT:PSS exhibits a pseudo capacitive behavior, its capacitance is constant in the working potential range and the distribution of the charge carriers concentration can be calculated from:

(3) $$\begin{array}{l} \rho \;\left(x\right)=\;\frac{C^{*}}{e}\left(E\left(x\right)-\;E_{o}\right) \end{array}$$

Where C^*^ is the volumetric capacitance (Proctor et al., 2016) and E(x) is the electrochemical potential at x point in the channel. E_o_ is the potential value, and thus the Fermi energy, above which the doping process of the polymer starts. It is an intrinsic feature of the material and can be evaluated by the onset potential in cyclic voltammetry (Cardona et al., 2011) or by the threshold E_g_ (Rivnay et al., 2015) in our device (E_Tg_). Considering the geometry of the device, E_o_ comes from:

(4) $$\begin{array}{l} E_{o}=\;E_{Tg}+\;\frac{V_{d}}{2} \end{array}$$

Therefore, by combining (1), (2), and (3) it results:

(5) $$\begin{array}{l} I_{d}=W\;d\;\mu \;C^{*}\left(E\left(x\right)-E_{o}\right)\frac{dE\left(x\right)}{dx} \end{array}$$

The integration of (5) must be performed considering the transistor geometry and the knowledge of the electrochemical potential of the gate electrode. Since the channel is symmetric with respect to the gate electrode, we can assume that V_d_ is equally distributed between the drain and source elements. Therefore, source and drain exhibit the potential of E_g_ – V_d_/2 and E_g_ + V_d_/2, respectively, and thus these values are used as boundary conditions for the integration. The resulting equation is:

(6) $$\begin{array}{l} I_{d}=\;\frac{W\;d}{L}\mu \;C^{*}\left(E_{g}-E_{o}\right)V_{d} \end{array}$$

Therefore, the E_g_ potential directly controls I_d_.

### Chloride Sensing

**Figure 3:**
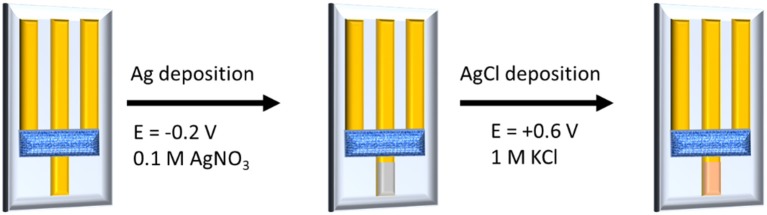
Gate modification by electrosynthesis steps.

For our devices, we have demonstrated that the gate voltage imposed by a potentiostat can control the electrical conductivity of the channel, and thus the device works as a transistor. If the gate electrode is modified with an active material, whose potential is controlled by the activity of a chemical species in solution, the potentiostat is no more necessary to force the gate potential and the OECT behavior depends on the chemical composition of the electrolyte. Electrochemical interfaces with a fixed and well-defined potential ruled by the activity of an anion in solution in equilibrium with sparingly soluble salts are called electrodes of second kind (Skoog et al., 1992). Ag/AgCl electrode, which is the most classic example of a second kind electrode, was chosen to modify our gate electrode. Ag/AgCl was obtained by two electrochemical steps (Figure 3). First, Ag was deposited on the bottom part of the gate gold track by applying a cathodic potential while the electrode was dipped in a 0.1 M AgNO_3_ solution. During the second step, an AgCl layer was formed by applying an anodic potential at the electrode immersed in a 1 M KCl solution. The deposition of Ag/AgCl layer was confirmed by Scanning Electron Microscopy (SEM) and Energy Dispersive X-ray Spectrometry (EDS) (Figures SI1, SI2). EDS analysis shows a prevalence of Ag, suggesting the presence of both Ag and AgCl materials that are necessary for the signal transduction.

**Figure 4:**
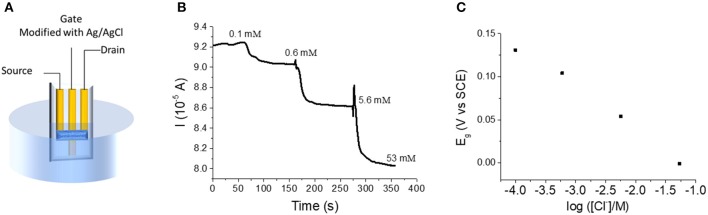
**(A)** Experimental setup for the OECT used as Cl^−^ sensor. **(B)** Id vs. time curve obtained for incremental Cl^−^ additions to the electrolyte solution (V_d_ = −0.01 V). **(C)** Trend of E_g_ following Cl^−^ additions.

The OECT was then tested as a sensor for chloride (Figure 3). The device was dipped in a 0.1 M KNO_3_ solution containing 1 × 10^−4^ M KCl under magnetic stirring (Figure 4A). While V_d_ was applied and I_d_ recorded, increasing amounts of KCl were added to the solution. Figure 4B shows the variation of I_d_ during the experiment. Each chloride addition leads to an I_d_ decrease. This result can be easily explained considering that an increase of Cl^−^ concentration generates a decrease of the gate potential value, as expressed by Nernst equation (Figure 4C). Since the electrical characterization of the OECT (transfer and output curve in Figure 2) clearly shows that an E_g_ decrease of the gate potential leads to a decrease of the I_d_, the device works on the basis of the transistor physics, as expected. Moreover, the drain current shows a linear dependence (I_d_ = −4.5 10^−6^ A decade^−1^ + 7.52 10^−5^ A) with the logarithm of Cl^−^ concentration, in agreement with Nernst equation. In fact, replacing E_g_ with the expression coming from Nernst equation for an Ag/AgCl electrode in (6), it results the response of the transistor as chemical sensor for chloride ions:

(7) $$\begin{array}{l} I_{d}=\;\frac{Wd}{L}\mu C^{*}\left(E_{AgCl/Ag}^{\Theta }-\;\frac{RT}{nF}\;ln\;a_{Cl^{-}}-\;E_{O}\right)V_{d} \end{array}$$

where a_Cl−_ is the activity of Cl^−^ ion in the solution and Θ is the standard potential of AgCl/Ag couple. The measurement is always carried out with an ionic strength buffer (0.1 M KNO_3_) to fix the activity coefficients, and thus to consider a logarithmic dependence on Cl^−^ concentration. The limit of detection is equal to 0.1 mM. Finally, it is worth calculating the lowest current resolution of the instrument in order to have a satisfactory precision on the concentration values. Considering both the sensitivity reported for our devices and the propagation of uncertainty, the resolution of current measurements should be lower than 10^−7^ A to have an error of ~4%.

The selectivity was also studied by adding solutions of different salts to the electrolyte (Figure SI3) while I_d_ was recorded. Li^+^, Na^+^, ${\text{ClO}}_{4}^{-}$, and ${\text{SO}}_{4}^{2-}$ presence does not alter the I_d_ value during the measurement because these anions do not react with Ag^+^ and therefore the redox equilibrium at the basis of the transistor operation is not modified. On the other hand, Br^−^ addition leads to an interference because the formation of AgBr consumes Ag^+^, thus changing E_g_ value and consequently the Fermi level of the gate electrode. The repeatability of the measurements was tested by constructing a random calibration plot three times (Figure SI4). For repeated measurement carried out at 3 10^−4^, 10^−3^, 3 10^−3^, and 10^−2^ M, the percentage standard deviations of the source-drain current value range between 0.04 and 1.6. However, the percentage error increases to 4-18% when data are expressed in terms of concentration. Finally, the signal stability was tested. Figure SI5 shows I_d_ recorded for a sensors dipped in a 0.1 KNO_3_ solution containing 10 mM KCl. After 10 h, I_d_ increased by 0.12%, which corresponds to a variation of the calculated concentration of 12%.

### Quantitative Measurements of Electrochemical Potentials

**Figure 5:**
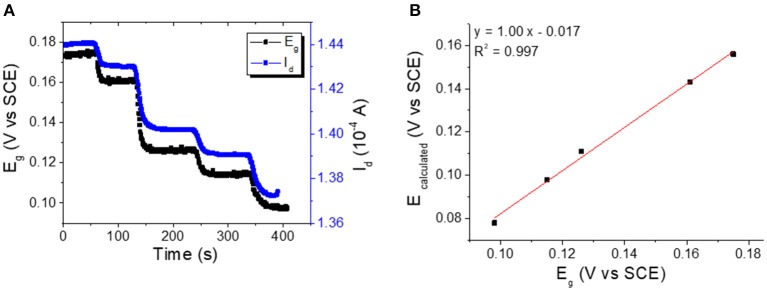
**(A)** E_g_ and I_d_ vs. time curves recorded for incremental Cl^−^ additions to the electrolyte solution (V_d_ = −0.01 V). **(B)** Plot of calculated vs. measured *E***-**Values.

In order to demonstrate the OECT working principle we measured the potential of the gate electrode during Cl^−^ sensing. This was accomplished by connecting the gate terminal to the potentiostat and measuring the open circuit potential between the gate and the reference electrode. Figure 5A shows I_d_ and E_g_ vs. time curves; as the two traces have the same trend it is obvious that a good correlation between the parameters exists. It is clear that the transistor response is ruled by a variation of the electrochemical potential of the gate electrode, but the open question is: “can the OECT quantitatively determine the electrochemical potential?” To answer this question, we calculated the E_g_ values from the measured I_d_ by using the transfer curve (Figure 2C) for each Cl^−^ concentration. The plot of calculated E_g_ vs. the corresponding measured E_g_ value (Figure 5B) shows a good linear correlation (*R*^2^ = 0.997) with a slope equal to 1.00 ± 0.03, and this confirms the ability of OECT in measuring the electrochemical potential. Being the intercept of the line −0.017 ± 0.004 V, it comes that there is a little systematic error in the estimation of E_g_ from the I_d_ value. This difference can be ascribed to the time shift between the transistor calibration, which must be performed before the gate electrode modification, and the actual measurement, which is carried out after the electrodeposition of Ag/AgCl on the gate. Although 17 mV is a huge error for a classical potentiometric measurement, each real-life application requires a calibration of the sensor, and the systematic errors can be easily overcome. The presented data clearly demonstrate the ability of our OECT device of measuring E_g_ without a reference electrode. A potentiometric transduction can be ascribed to our sensors also from a rigorous electrochemical point of view.

### Iodide, Bromide, and Sulfide Sensing

The working principle described for the Cl^−^ detection can be extended to other species. The gate electrode can be easily modified with Ag/AgBr, Ag/AgI, or Ag/Ag_2_S to produce sensors able to detect bromide, iodide or sulfide, respectively. The fabrication is very simple and requires only a change of the electrolyte during the second step of the gate modification (Figure 3). The sensors were tested with the same procedure used for chloride, and as already observed, the addition of the analyte led to a decrease of the drain current proportional to the logarithm of the concentration. In order to compare the analytical performances of the proposed sensors, a normalization of the current is required due to the high variability of the background values associated to our devices. The current was normalized (I_N_) on the basis of the highest value observed during the experiments, i.e., when the concentration of the target ion was 0.1 mM, according to:

$$\begin{array}{l} I_{N}=1-\frac{I_{d}}{I_{dmax}}=1-\frac{E_{AgX/Ag}^{\Theta }-\frac{RT}{nF}\;ln\;a_{X^{n-}}-E_{O}}{E_{AgX/Ag}^{\Theta }-\frac{RT}{nF}\text{ ln }a_{X_{\text{min}}^{n-}}-E_{O}} \end{array}$$

The normalized sensitivity of the devices, which is the variation of the analytical signal as a function of the logarithm of analyte concentration, can be expressed as:

$$\begin{array}{l} s_{N}=\;\frac{dI_{N}}{dlna_{X^{n-}}}=\frac{RT}{nF\left(E_{AgX/Ag}^{\Theta }-\frac{RT}{nF}\text{ ln }a_{X_{\text{min}}^{n-}}-E_{O}\right)} \end{array}$$

**Figure 6:**
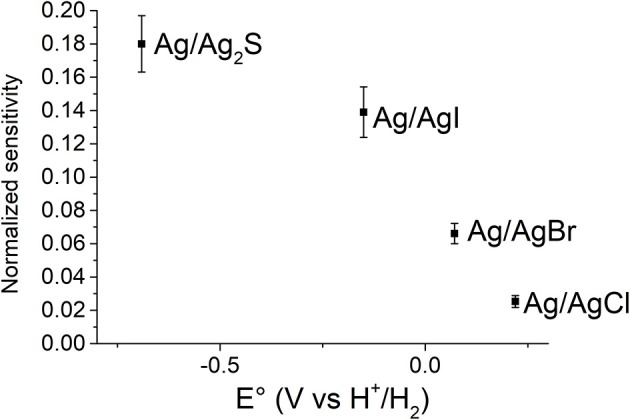
Normalized sensitivity vs. standard potential of gate electrodes embedded in the OECT.

Consequently, the sensitivity becomes higher by decreasing the difference between $E_{AgX/Ag}^{\Theta }$ and $\frac{RT}{nF}\ln a_{X_{\min}^{n-}}+E_{O}$, and thus the highest sensitivity is obtained for the sensor endowed with the gate electrode modified with the Ag/Ag_n_X interface whose standard potential is closest to the E_O_ value. This trend is well-evident from Figure 6, which shows the normalized sensitivity as a function of the standard potential of the gate electrode modifiers. Although the normalization is used for comparison aims, the normalized sensitivity actually conveys a real sensing performance. Since the OECT works in depletion mode, the analytical signal is a current decrement. Therefore, the higher the normalized sensitivity, the higher the percentage current variation and, thus the ability to discriminate two close concentration values. Moreover, the error associated to the concentration values is lower if the normalized sensitivity is higher, because the noise is a constant I_d_ fraction. These results show that the best performance can be reached by combining the electrical features of the organic semiconducting material and the gate electrode modifier. As previously reported in literature (Kergoat et al., 2012) the gate material affects the transistor operation and it can be exploited in chemical sensing (Tarabella et al., 2010). The OECT should work at an electrochemical potential that is close to the onset potential of the conductive polymer.

### OECT as Amplifier

**Figure 7:**
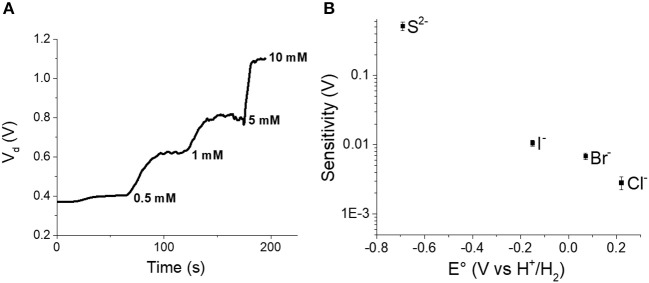
**(A)** V_d_ obtained for incremental S^2−^ additions to the electrolyte solution. **(B)** Sensitivity calculated in current-driven mode vs. standard potential of gate electrodes embedded in the OECT.

One of the advantages of the transistor architecture is the possibility of amplifying signals, and this feature can be useful to overcome the thermodynamic limit represented by Nernst equation. A transistor could be integrated in an appropriate electrical circuit in order to reach the best performance. However, we have tried to exploit our OECT as amplifier in current-driven mode by measuring the V_d_ necessary to sustain the flow of a fixed I_d_ (1 mA) in the channel, while the analyte concentration was varied in the electrolyte (Figure 7A).

Each analyte addition leads to a V_d_ increase because both the electrochemical potential of the gate electrode and the PEDOT:PSS conductivity decrease and thus a higher V_d_ is required to guarantee a I_d_ equal.

Similarly to other OECTs setup, the relationship that links V_d_ to ${\text{a}}_{\text{X}}^{n-}$ is:

$$\begin{array}{l} V_{d}=\;\frac{I_{d}L}{Wd\mu C^{*}\left(E_{AgX/Ag}^{\Theta }-\frac{RT}{nF}\;ln\;a_{X^{n-}}-E_{O}\right)} \end{array}$$

Although this relationship is not linear, considering that V_d_ varies in a small range, we can approximate the sensor response to a line as in the first term of a Taylor series. In such a way, it is possible to make a comparison with the values expected from the Nernst equation in order to experimentally verify an amplification due to transistor architecture. The Pearson coefficients were always higher than 0.93 confirming that the trends are not actually linear, but the approximation can be accepted for comparison aims. Figure 7B shows the sensitivities values obtained for the different analytes as a function of the standard potential of the gate electrode. Also in this case, the value of the standard potential plays a key role in the performance of the device. The closer the standard potential to the E_o_, the higher the sensitivity. In fact, we can overrun the Nernstian behavior only for S^2−^ anion as its standard potential is very close to E_O_. Forcing the current to 1 mA enables a sensitivity equal to 0.52 V decade^−1^ that is more than 17 times higher than the sensitivity expected from the Nernst equation.

## Discussion

### Transistor Architecture

The results reported in this paper show that an OECT can measure an electrochemical potential and thus it can be used to acquire potentiometric signals. It may seem obvious that the variation of the gate voltage can modulate the output I_d_ in a transistor. Among electrolyte-gated organic transistors, the potential is usually applied with respect to the source terminal, which is grounded. The threshold voltage or I_d_ variation are widely used as analytical signals in the transistor community. Nevertheless, in electrochemistry all potentials are referred to a reference electrode so as to be linked to the Fermi level and to the equilibrium position of chemical reactions at the basis of the transduction process. The knowledge of the electrochemical potential allows the use of all the electrochemical equations that are at the basis of sensing. It should be emphasized that a multitude of electrochemical sensors has been developed and they could be used as transducer embedded in a transistor architecture. The direct measurement of electrochemical potentials facilitates their integration in a transistor and the understanding of the processes at the basis of sensing. In a discussion concerning the measurement of electrochemical potential with a transistor, it should be pointed out that ISFETs are usually considered potentiometric sensors. However, the reference electrode is a key component in ISFETs' architecture and thus they suffer the same disadvantages described in the introduction about their miniaturization and fabrication. Moreover, ISFETs measure an interface potential that exhibits a sensitivity which is lower or at least equal to Nernstian value. In fact, the chemistry associated to the transduction usually leads to a lower sensitivity that can be described considering surface equilibria and surface ions concentrations.

For the indirect measurement of E_g_, a configuration that is very close to the one proposed by Wrigton's group (Kittlesen et al., 1984; Paul et al., 1985) has been exploited. Although it was reported in literature 30 years ago, most papers report an architecture with a gate electrode that is not in electrical contact with the channel, but only with the electrolytic solution. For this reason, it is worth discussing the difference between the configurations. The key feature of our device is the ability of controlling the channel electrochemical potential by the gate electrode that is in direct contact with the channel. In this way, each electrode whose potential is linked to the concentration of a target species in solution, could be used as transducer to produce a chemical sensor. Since a large number of potentiometric sensors have been described in the literature, the here proposed OECT architecture could pave the way to the development of new sensors with boosted performance. Obviously, some constraints could hinder the real development. For example, the gate electrode must be able to change the charge carriers concentration in the semiconducting material because, from an electronic point of view, the gate capacitance must be higher than the one of the channel. For this reason, a non-polarizable electrode, such as that used in this paper, should ideally guarantee the best performance.

An advantage of our sensor is the ability of working as a two-terminal device as the modified gate electrode establishes a fixed potential due to an electrochemical process that is similar to those occurring in a battery. The gate electrochemical interface generates an electromotive force that polarizes also the channel when the equilibrium is reached. Consequently, a gate terminal connected to the read-out electronics is no more necessary. The only precondition that is mandatory for sensing is the direct electrical contact between the sensing material of the transducer and the conductive polymer. For example, Kim et al. (2018) fabricated an OECT based sensor for Cl^−^ on a textile by a simple short-cut of the silver/silver chloride wire with a PEDOT yarn. Moreover, when this principle was applied at a nanoscale level with the intercalation of silver/silver chloride nanoparticles into a conductive polymer, the new composite was exploited for the fabrication of a chloride sensor (Gualandi et al., 2018b). The proposed approach is easily applicable to unconventional substrates, e.g., a textile wire, in order to produce wearable sensors with an important simplification of the architecture. For such sensors, the electrolyte solution consists of a matrix that contains the target molecules, and it is not necessary for transmitting the electrical action of the gate on the conductive polymer. Although the two-terminal configuration significantly simplifies the sensor fabrication, it does not allow for the control of the channel conductivity to optimize the sensing performance. In fact, Ghittorelli et al. (2018) were able to achieve a high amplification of the potentiometric signal by varying the gate potential using a three terminal architecture. On the other hand, the direct control of channel electrochemical potential cannot be accomplished with this configuration. Furthermore, the V_g_ application determines an ionic current in the electrolyte and does not allow reaching the thermodynamic equilibrium, with a consequent lower stability of the signal. A more complex architecture has been proposed to reach stability and reproducibility by Malliaras' group (Scheiblin et al., 2017), consisting in an OECT with a gate modified with iridium oxide which was connected with a similar OECT by a Wheatstone bridge. This configuration is quite complex and can be hardly used in more advanced devices such as wearable sensors and lab on a chip applications.

Finally, it is worth noting that our configuration is particularly suitable for the acquisition of potentiometric signals when we can speculate that an amperometric transduction, like that based on a Faradaic reaction mediated by an enzyme, would be more difficult. As an example, Bartlett and Birkin (1993) described a two-terminal device based on an amperometric transduction which required a pre-polarization of the channel with a potentiostat. The transduction occurred thanks to the reaction between a redox mediator which shuttled charges between the conducting polymer (polyaniline, PANI) and the enzyme glucose oxidase, after the oxidation of glucose to gluconolactone, but the oxidized centers in PANI had to be created applying an anodic potential before each determination.

In conclusion, our architecture is particularly suitable for a potentiometric detection, while an amperometric transduction can be easily performed with OECTs endowed with a gate electrode which is separated from the channel. It is worth underlining that the potentiometric transduction is usually more robust than the amperometric one because it depends only on the thermodynamics of the system, while both thermodynamics and kinetics of the electrochemical reactions affect an amperometric transduction. It follows that such a control in a transistor operating with an amperometric transduction is hardly achievable because of the lack of a reference electrode and of the presence of a leakage current flowing through the electrolyte.

### OECT as a Tool for Measuring Potential

The performance of our OECT can be compared with other tools for monitoring potentiometric signals. The reference electrode is indispensable for the measurement of potentials generating at liquid/solid with an indicator electrode or a ISFET. The classical reference electrodes exhibit the best features; however, the limits described in the introduction hinder their miniaturization and use in the most advanced applications (Sophocleous and Atkinson, 2017). The literature (Sophocleous and Atkinson, 2017; Cuartero and Crespo, 2018; Parrilla et al., 2019) suggests screen-printed electrodes, mainly based on the Ag/AgCl electrode, to solve these drawbacks. Although the quasi-reference electrodes are the most popular in the panorama of electrochemical sensors, their real use should be limited to the amperometric sensors due the variability of their potential. Therefore, the screen-printing of a full reference electrode is the only option for the production of potentiometric sensors. Since these electrodes have been produced since the late 90's (Desmond et al., 1997; Mroz et al., 1998) a good degree of optimization has been reached, even if they are not ready for a reliable commercialization. Consequently, considering the different technology maturities of the OECTs for a direct potentiometric measurement and the screen-printed reference electrodes, a direct comparison is difficult, even if some OECTs advantages are clearly visible. In addition, potentiometric measurements can be performed for applications that range from disposable devices to long term monitoring and, thus, different parameters play key roles in the definition of their performance.

It is undeniable that the architecture of a common potentiometric sensor requires a very low energy consumption to perform the measurement. On the other hand, our OECTs offer some intriguing features. The main advantages are: (i) the easy fabrication of the channel that is the only element required for the potential measurement as only the PEDOT:PSS channel, and its electrical contact, must be printed. On the contrary, the full screen printed reference electrodes are composed of at least three layers, one consisting of a gel to fix the ion concentration, but the ion leakage can really limit their stability; (ii) the amplification of the signal can lead to sensitivities higher than the thermodynamic limit of the Nernst equation. Moreover, it is worth noting that the sensitivity of the OECT working as a potentiometric sensor can be increased by varying both the geometry of the device and the I_d_. This is not possible for the classical potentiometric sensor whose response is simple controlled by thermodynamics.

Summarizing, the here-proposed OECTs can measure potentiometric signals and represent a viable option to design and fabricate new devices for advanced sensing applications (wearable, lab-on-a-chip and so on) thanks to thorough knowledge of the electrochemical interfaces involved in the generation of OECT signal. Replacing the reference electrode with a PEDOT:PSS channel significantly simplifies sensor miniaturization, increases the compatibility with flexible substrates and reduces the production cost. In addition, the sensor performance can be boosted by an optimization of the devices in terms of geometry and employed materials as the transistor architecture can amplify the signal, thus overcoming the Nernstian limit.

## Data Availability Statement

The datasets generated for this study are available on request to the corresponding author.

## Author Contributions

IG wrote the manuscript text and equations to explain the device working principle. IG, FM, and MT carried out the experiments which are reported in the manuscript. IG and MT designed the new transistor architecture. DT positioned the experimental results in analytical chemistry background. BF and ES coordinated the research. ES, BF, and DT supervised the research. All authors reviewed the manuscript.

### Conflict of Interest

The authors declare that the research was conducted in the absence of any commercial or financial relationships that could be construed as a potential conflict of interest.
