# Supplementary material for: Organic Electrochemical Transistors as Versatile Analytical Potentiometric Sensors
Source: Front Bioeng Biotechnol. 2019 Nov 22;7:354. doi: 10.3389/fbioe.2019.00354 (PMC6882742; doi:10.3389/fbioe.2019.00354)
Supplement: Supplementary file 1 [file Table_1.DOCX]

Supplementary materials

Organic electrochemical transistors as versatile analytical potentiometric sensors

Isacco Gualandi^1*^, Marta Tessarolo^2^, Federica Mariani^1^, Domenica Tonelli^1^, Beatrice Fraboni^2^, Erika Scavetta^1*^

^1^Dipartimento di Chimica Industriale ‘Toso Montanari’, Università di Bologna, Viale Risorgimento 4, 40136 Bologna, Italy

^2^ Dipartimento diFisica e Astronomia, Università di Bologna, Viale Berti Pichat 6/2, 40127 Bologna, Italy


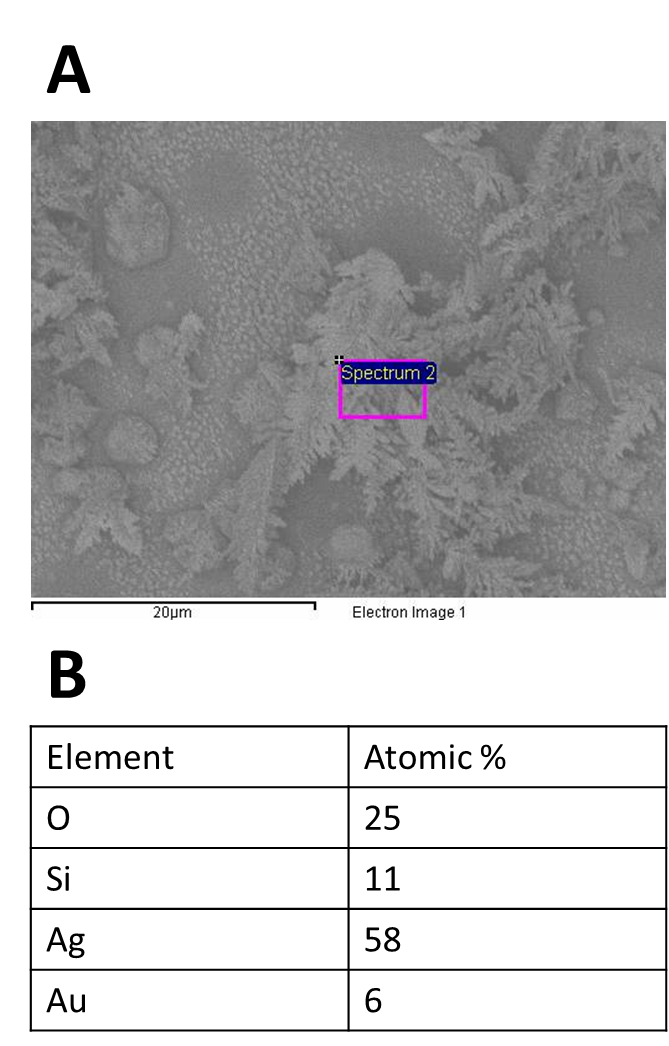


**Figure SI 1.** A) SEM image of silver electrodeposited on the gold gate electrode. B) Elemental analysis of the electrodeposited layer.

**
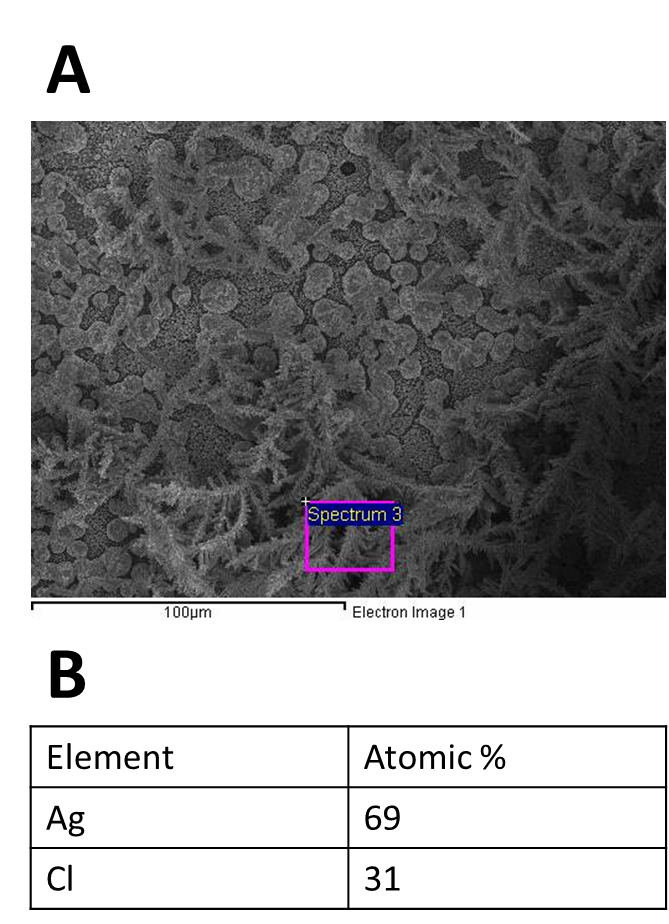
**

**Figure SI 2.** A) SEM image of silver/silver chloride electrodeposited on the gold gate electrode. B) Elemental analysis of the electrodeposited layer.

**Fig SI 3.** I_d_ *vs* time (V_d_ = 0.01 V) recorded while different compounds at 1 mM concentration were added to the electrolyte (0.1 M KNO_3_).

**Fig SI 4.** Repeatability of the proposed sensor (V_d_ = 0.01 V) evaluated by performing the measurements in unstirred solutions containing different Cl^-^ amounts (0.3 mM, 1 mM, 3 mM, and 10 mM). The measurements are randomly repeated 3 times.

**Fig SI 5.** Id *vs* time curve recorded in a 0.1 M KNO_3_ solution containing 10 mM Cl^-^.
